# Siltuximab downregulates interleukin-8 and pentraxin 3 to improve ventilatory status and survival in severe COVID-19

**DOI:** 10.1038/s41375-021-01299-x

**Published:** 2021-05-24

**Authors:** Giuseppe Gritti, Federico Raimondi, Barbara Bottazzi, Diego Ripamonti, Ivano Riva, Francesco Landi, Leonardo Alborghetti, Marco Frigeni, Marianna Damiani, Caterina Micò, Stefano Fagiuoli, Ferdinando Luca Lorini, Lucia Gandini, Luca Novelli, Jonathan P. Morgan, Benjamin M. J. Owens, Karan J. K. Kanhai, Gordana Tonkovic Reljanovic, Marco Rizzi, Fabiano Di Marco, Alberto Mantovani, Alessandro Rambaldi

**Affiliations:** 1 Hematology Unit, Azienda Socio Sanitario Territoriale Papa Giovanni XXIII, Bergamo, Italy; 2 Pneumology Unit, Azienda Socio Sanitario Territoriale Papa Giovanni XXIII, Bergamo, Italy; 3 Postgraduate School of Respiratory Medicine, University of Milan, Milan, Italy; 4 Humanitas Clinical and Research Center – IRCCS, Milan, Italy; 5 Infectious Diseases Unit, Azienda Socio Sanitario Territoriale Papa Giovanni XXIII, Bergamo, Italy; 6 Intensive Care Unit, Azienda Socio Sanitario Territoriale Papa Giovanni XXIII, Bergamo, Italy; 7 Postgraduate School of Hematology, University of Milan, Milan, Italy; 8 Postgraduate School of Anesthesiology and Intensive Care, University of Milan, Milan, Italy; 9 Gastroenterology Unit, Azienda Socio Sanitario Territoriale Papa Giovanni XXIII, Bergamo, Italy; 10 EUSA Pharma, Hemel Hempstead, UK; 11 ErgoMed PLC, Guildford, UK; 12 Department of Health Sciences, University of Milan, Milan, Italy; 13 Department of Biomedical Sciences, Humanitas University, Milan, Italy; 14 The William Harvey Research Institute, Queen Mary University of London, London, UK; 15 Department of Oncology-Hematology, University of Milan, Milan, Italy

**Keywords:** Infectious diseases, Medical research, Infectious diseases

## To the Editor:

Severe coronavirus disease 2019 (COVID-19) is characterized by interstitial pneumonia/acute respiratory distress syndrome and hyperinflammation, with elevated levels of proinflammatory cytokines, such as interleukin-6 (IL-6), associated with mortality and patients requiring ventilator support [1–3]. Targeting the IL-6 signaling pathway has been identified as a potential strategy to mitigate the elevated cytokines and resulting hyperinflammation associated with COVID-19 [1]. Siltuximab is the first and only US Food and Drug Administration- and European Medicines Agency-approved monoclonal antibody that specifically binds to IL-6, thereby inactivating IL-6–induced signaling. Siltuximab is currently approved for the treatment of adults with idiopathic multicentric Castleman disease [4]. The aim of this study was to examine the association between siltuximab treatment, serum cytokine and chemokine levels, and mortality and/or respiratory function in hospitalized patients with COVID-19 and acute respiratory distress syndrome.

We designed a prospective, observational cohort study at the start of the pandemic in response to the urgent unmet need for an effective treatment for patients with SARS-CoV-2 pneumonia, hyperinflammation, and respiratory failure [5]. In accordance with clinical guidelines developed at the Papa Giovanni XXIII Hospital in Bergamo, Italy, siltuximab was initially supplied under a compassionate-use program for the emergency treatment of 30 patients with severe COVID-19 requiring ventilatory support. Consequently, an investigator-initiated study protocol was developed for immediate implementation. The study protocol was submitted and approved through the Hospital Ethics Board. Patients, or their legal representative, provided either verbal or written consent to participate in the study. The inclusion and exclusion criteria are detailed in the Supplementary Materials and Methods.

All patients were monitored according to the hospital and Italian national guidelines for a minimum of 30 days, and if a patient was discharged from the hospital, they were asked to provide relevant laboratory results and safety information for 30 days following the start of treatment. The primary endpoint of this study was mortality in COVID-19 patients treated with siltuximab, calculated as the time from ventilation to death from any cause within 30 days of treatment [6]. As part of the exploratory analyses, we wanted to assess the prognostic effects of the down-modulation of cytokines by siltuximab. For this reason, we explored the change in cytokine levels and ventilatory status within 30 days of treatment and patient mortality. In addition, the ventilatory support parameters, respiratory function (ratio of arterial oxygen partial pressure to fractional inspired oxygen), and adverse events according to the US National Cancer Institute Common Terminology Criteria for Adverse Events v4.03, within 30 days of siltuximab treatment, were evaluated.

Data were obtained from hospital medical records and included demographic data, presenting symptoms and history of previous treatments, vital signs, ventilatory support, and laboratory data, including blood counts and AST, ALT, creatinine, procalcitonin, lactate dehydrogenase, and CRP levels. The following cytokines and chemokines were measured on days 1 and 4 of the study: pentraxin 3 (PTX3), IL-8, IL-10, IL-12, TNF, CXCL10/IP-10, CXCL9/MIG, CCL2/MCP-1, and sCD163 (details in Supplementary Materials and Methods). Response to treatment was defined as a reduction in the need for ventilatory support and the resolution of symptoms and signs of COVID-19.

Patients received a siltuximab dose of 11 mg/kg administered intravenously over an hour. A second dose was permitted 72 h after the first dose at the physician’s discretion. Standard treatment was provided according to hospital guidelines (detailed in the Supplementary Materials and Methods).

Forward stepwise regression modeling was performed to assess the association of cytokines with mortality status and ventilatory outcomes (details in Supplementary Materials and Methods). A *p* value of ≤ 0.05 was considered significant.

**Fig. 1:**
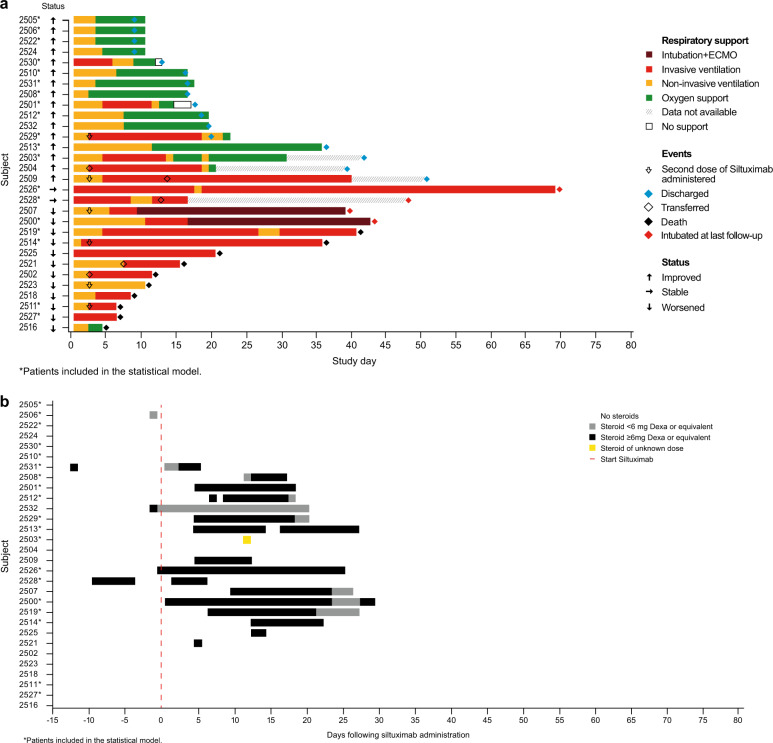
Patient outcome and corticosteroid use. **a** Individual patient outcomes following treatment with siltuximab. Changes in oxygen support and clinical outcome from day 1 through study completion for all siltuximab-treated patients (*N* = 30). *EMCO* extracorporeal membrane oxygenation. Patients who needed to be in an ICU were transferred to another hospital. The four patients who were still intubated at last follow-up time are alive. **b** Corticosteroid use. Steroid use in relation to starting siltuximab (*N* = 30). *Dexa* dexamethasone.

The baseline characteristics and hematological parameters of patients treated with siltuximab are provided in the Supplementary Results (Supplementary Tables 1 and 2). Of the 30 patients treated with siltuximab, 10 died, the condition of four patients either remained the same or deteriorated, and 16 showed improvements in ventilatory status and were discharged from the hospital (Fig. 1a). Twelve of the 30 siltuximab-treated patients did not receive corticosteroids at any time during the study period. Corticosteroids were intravenously administered to the other 18 siltuximab-treated patients. Of these 18, five patients received corticosteroids prior to siltuximab treatment, while 13 patients received corticosteroids after day 4 and, therefore, after siltuximab treatment. Three patients received corticosteroids only for 1 day, of which only one had them administered prior to siltuximab treatment (Fig. 1b).

Ten patients who had concomitant siltuximab and corticosteroids improved and were discharged. Two patients remained stable, and six patients died. Of patients who had siltuximab and corticosteroid dosing for longer than 5 days, seven had improved ventilatory status and survival, the condition of two patients remained stable, and four died (Fig. 1b).

**Fig. 2:**
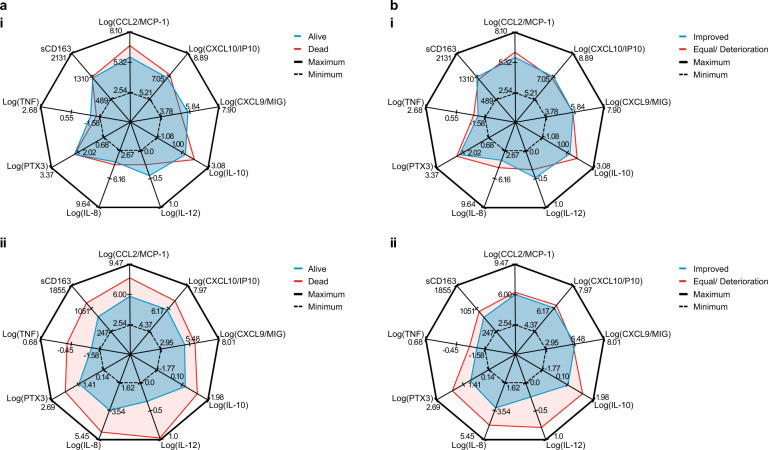
Radar plot showing association between cytokines and mortality/ventilatory outcomes. **a** Mortality outcome. Radar plots for the various covariates of the day 1 (before treatment [i]) and day 4 (ii) median values of patients who responded to treatment (green) or had died (red) by day 30. The axis/spokes represent the minimum and maximum values in the day 1 and day 4 datasets (*n* = 22). Higher levels of blood IL-8, IL-12, sCD163, CCL2, and TNF on day 4 were associated with mortality at day 30 (*p* < 0.05). **b** Ventilatory outcome. Radar plots for the various covariates of the day 1 (before treatment [i]) and day 4 (ii) median values of patients who responded to treatment and no longer required ventilatory support (green) or remained on ventilatory support (red) by day 30. The axis/spokes represent the minimum and maximum values in the day 1 and day 4 datasets (*n* = 22). Patients with lower levels of PTX, IL-8, IL-10, and IL-12 measured on day 4 had improved ventilatory status at day 30.

Baseline levels of blood cytokines and chemokines (PTX3, IL-8, IL-10, IL-12, CRP, sCD163, CXCL10, CCL2, CXCL9, and TNF) were neither correlated with nor predictive of mortality (Fig. 2a) or ventilatory status (Fig. 2b) on day 30.

Univariate analysis of low compared with high levels of blood IL-8 (*p* = 0.0099), IL-12 (*p* = 0.0154), sCD163 (*p* = 0.0437), CCL2/MCP-1 (*p* = 0.0303), and TNF (*p* = 0.0145) measured on day 4 post siltuximab treatment suggested that lower levels were associated with better survival at day 30 (Fig. 2a). However, IL-8 (*p* = 0.050) was the only cytokine that was significantly associated with better survival when using the multivariate stepwise regression model. In the predictive model (Supplementary Fig. 1a), patients with IL-8 levels within the lower quartile range (Q1) had a probability of 99.7% of being alive (95% confidence interval [CI] 62.1–99.7), while patients with IL-8 levels within the upper quartile range (Q3) had a decreased probability of 0.788 of being alive (95% CI 42.5–94.9).

The univariate analysis identified that patients with lower levels compared with higher levels of PTX3 (*p* = 0.0056), IL-8 (*p* = 0.015), IL-10 (*p* = 0.0196), and IL-12 (*p* = 0.0113) measured on day 4 following siltuximab treatment had improved ventilatory status at day 30 (Fig. 2b). However, stepwise regression modeling only retained PTX3 (*p* = 0.051) and IL-8 (*p* = 0.061) as cytokines that were associated with improved ventilatory status. In the predictive model, patients with PTX3 levels within Q1 and IL-8 levels within median levels had a 92.0% probability of improvement in ventilatory status (95% CI 41.7–99.5), while this dropped to 89.2% for patients with PTX3 within median levels and IL-8 within Q1 (95% CI 43.3–98.9). Meanwhile, patients with PTX3 within Q3 and median IL-8 had just a 27.8% probability of improvement in ventilatory status (95% CI 7.3–65.5). Similarly, patients with median PTX3 and IL-8 within Q3 had a 40.6% probability of improvement in ventilatory status (95% CI 13.1–75.6; Supplementary Fig. 1b).

The multivariable stepwise regression model was able to correctly predict outcomes in 19/22 (86%) and 20/22 (91%) patients for day 30 mortality and day 30 ventilatory status, respectively (Supplementary Table 3).

Adverse events were recorded for all 30 siltuximab-treated patients included in the study. No new or unexpected drug-related adverse events were reported in siltuximab-treated patients, and the majority were Grade 3 or below (Supplementary Tables 4 and 5).

To our knowledge, this is the first study to report on treatment with siltuximab in patients with COVID-19 and to show that modification of the cytokine profile after treatment is prognostic of outcome. Our data suggest that a timely modulation of PTX3 by siltuximab represents a local effect of IL-6 inhibition in reducing inflammation in the lung bronchoalveolar lavage fluid [7, 8]. Moreover, modulation of IL-8 levels by siltuximab suggests an inhibition of systemic inflammation, which may prevent IL-8–mediated recruitment and activation of neutrophils [9, 10]. The beneficial effects of siltuximab in reducing local and systemic inflammation are reflected in the improved survival and respiratory function in patients with severe COVID-19 who had attenuated cytokine storms. We hypothesize that patients who do not exhibit a response to siltuximab-mediated inhibition of the cytokine storm are less likely to show improvements in response to severe COVID-19 infection without a change in management strategy. Siltuximab was well tolerated, and the adverse event profile in patients with COVID-19 was similar to that reported for patients with idiopathic multicentric Castleman disease [11]. There were no adverse events that were related to siltuximab.

Our study has limitations. It is an observational study with a small sample size and no control patients, which limits the interpretation and generalizability of the results. Unlike multivariable analysis, univariate analysis cannot examine relationships between different factors. However, careful consideration is required for the interpretation of the multivariable analyses performed on a small sample size. Given the study’s limitations, the results require a comprehensive assessment in adequately powered randomized controlled trials.

In conclusion, this preliminary study suggests that the reduction of IL-8 and PTX3 levels on day 4 following siltuximab treatment is associated with improved survival and ventilatory outcomes in patients hospitalized for COVID-19. A randomized clinical trial would be informative to confirm the efficacy and safety of this IL-6–neutralizing monoclonal antibody in the treatment of patients with viral acute respiratory distress syndrome.

## Supplementary information

Supplementary Information
