# Supplementary material for: Siltuximab downregulates interleukin-8 and pentraxin 3 to improve ventilatory status and survival in severe COVID-19
Source: Leukemia. 2021 May 24;35(9):2710–4. doi: 10.1038/s41375-021-01299-x (PMC8142063; doi:10.1038/s41375-021-01299-x)
Supplement: Supplementary file 1 — Supplementary Information [file 41375_2021_1299_MOESM1_ESM.docx]

**Siltuximab downregulates interleukin-8 and pentraxin 3 to improve ventilatory status and survival in severe COVID-19**

Giuseppe Gritti, Federico Raimondi, Barbara Bottazzi, Diego Ripamonti, Ivano Riva, Francesco Landi, Leonardo Alborghetti, Marco Frigeni, Marianna Damiani, Caterina Micò, Stefano Fagiuoli, Ferdinando Luca Lorini, Lucia Gandini, Luca Novelli, Jonathan P. Morgan, Benjamin M. J. Owens, Karan J. K. Kanhai, Gordana Tonkovic Reljanovic, Marco Rizzi, Fabiano Di Marco, Alberto Mantovani, Alessandro Rambaldi

**SUPPLEMENTARY MATERIAL**

**SUPPLEMENTARY MATERIALS AND METHODS**

**Inclusion and exclusion criteria for the study**

Consecutive patients with COVID-19 and interstitial pneumonia, who were hospitalized and required ventilatory support by either invasive mechanical ventilation (IMV), non-invasive ventilation (NIV), or continuous positive airway pressure (CPAP), and who met the following criteria were included in the study: diagnosis of pulmonary infection with SARS-CoV-2, confirmed by a reverse transcriptase quantitative polymerase chain reaction assay, and acute respiratory distress syndrome in accordance with the Berlin 2012 criteria [1].

Patients with an active bacterial or viral (not SARS-CoV-2) pulmonary infection not controlled by treatment were excluded from the study, and those treated with other anti-interleukin therapies, including IL-6 receptor blockers, were not eligible to participate. Patients treated with siltuximab were grouped according to the type of ventilatory support received at the start of siltuximab treatment: patients receiving CPAP/NIV and patients receiving IMV.

**Measurements of cytokines and chemokines**

An enzyme-linked immunosorbent assay kit was used to quantify sCD163 according to the manufacturer’s instructions (R&D Systems Inc., Minneapolis, MN, USA). Inflammatory cytokines (IL-8, IL-10, IL-12, TNF) and chemokines (CXCL10/IP-10, CXCL9/MIG, CCL2/MCP-1) included were dosed in a single serum sample by flow cytometry (BD CBA Human Inflammatory Cytokines Kit, BD Biosciences, San Jose, CA, USA). PTX3 plasma levels were measured, as previously described [2], by a sandwich enzyme-linked immunosorbent assay (detection limit 0.1 ng/mL, inter-assay variability 8–10%) developed in house. The inflammatory parameters were measured on days 1 and 4 after the start of siltuximab treatment. The personnel who conducted the tests were blinded to patients’ characteristics.

**Standard treatments for COVID-19**

Standard treatments were administered according to the hospital guidelines and included antiviral therapy (administration was influenced by drug availability and included lopinavir/ritonavir 200/50 mg two tablets twice daily, darunavir/cobicistat 800/150 mg one tablet once daily, or remdesivir 200 mg administered intravenously on day 1, followed by 100 mg daily for the remaining 9 days of treatment), and hydroxychloroquine 200 mg twice daily. Steroid use was not permitted according to local guidelines until 27 March 2020, when high-dose corticosteroids (intravenous methylprednisolone 1 mg/kg daily for 5 days or equivalent dose of oral prednisone, both followed by tapered doses) were added to the treatment guidelines. Similarly, after that date, subcutaneous prophylactic low molecular weight heparin (4000 IU once daily) was introduced in all patients at admission.

**Statistical analyses**

Cytokine data at days 1 and 4 were explored using descriptive statistics, and all variables except sCD163 were log-transformed to satisfy the conditions necessary to apply the regression models. Not all the inflammatory parameters were determined at days 1 and 4. Since no valid method was available to replace the missing values, statistical modeling was performed for 22 of the 30 patients included in the study. Eight patients for whom cytokine levels on days 1 and 4 were not measured were excluded from this analysis.

A univariate approach was used to investigate the effect of all biological parameters (day 1, day 4) on the mortality status and ventilatory status on day 30. The parameters that surpassed the univariate significance of *p* ≤ 0.05 were selected for the forward stepwise regression model and associated with mortality status and ventilatory outcomes.

All statistical analyses were performed using SAS Version 9.4 software (SAS Institute, Inc., Cary, NC, USA). A *p*-value ≤ 0.05 was considered statistically significant. As this was an exploratory study, correction for the multiple testing was not performed.

**SUPPLEMENTARY RESULTS**

**Patient characteristics**

Thirty patients with COVID-19 confirmed by a nasopharyngeal swab positive test for SARS-CoV-2 RNA and respiratory failure requiring ventilatory support were treated with siltuximab between 7 March and 9 April 2020. The baseline patient characteristics and hematological parameters are shown in Supplementary Tables 1 and 2, respectively.

The majority of siltuximab-treated patients were receiving CPAP/NIV at the time of treatment (Supplementary Table 2). They were given a single dose of siltuximab within 48 hours of initiating ventilatory support: 10 patients received siltuximab on the same day as CPAP, nine patients received siltuximab the day after CPAP, five patients received siltuximab 2 days after CPAP, and one patient received siltuximab 13 days after CPAP. Five patients required IMV before siltuximab treatment. Of these patients, four progressed from CPAP/NIV to IMV before siltuximab treatment, and one required IMV upon admission and was then treated with siltuximab. Six patients received a second dose 72 hours after the first dose of siltuximab (Fig. 1a).

**References**

1. Ranieri VM, Rubenfeld GD, Thompson BT, Ferguson ND, Caldwell E, Fan E, et al. Acute respiratory distress syndrome: the Berlin Definition. JAMA. 2012;307:2526–33.
2. Brunetta E, Folci M, Bottazzi B, De Santis M, Gritti G, Protti A, et al. Macrophage expression and prognostic significance of the long pentraxin PTX3 in COVID-19. Nat Immunol. 2021;22:19–24.


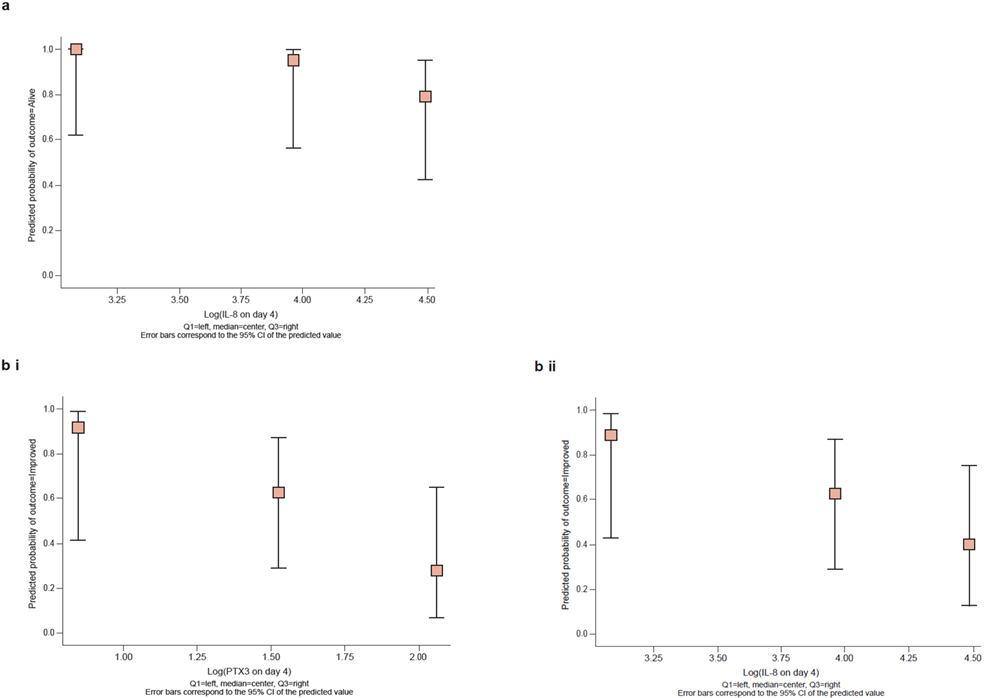


**Supplementary Fig. 1** **Predicted probabilities of mortality and ventilatory outcomes. A** Predicted probability of being alive based on the levels of IL-8 measured on day 4. **B** Predicted probability of having an improved ventilatory outcome based on the levels of PTX3 (i) and IL-8 (ii) measured on day 4.

**Supplementary Table 1** Baseline patient and disease characteristics for siltuximab-treated patients.

|  | Siltuximab-treated patients, CPAP/NIV (*n* = 25) | Siltuximab-treated patients,  IMV (*n* = 5) | All siltuximab patients (*N = 30*) |
| --- | --- | --- | --- |
| Sex |  |  |  |
| Male | 19 (76.0%) | 4 (80.0%) | 23 (76.6%) |
| Female | 6 (24.0%) | 1 (20.0%) | 7 (23.3%) |
| Age, years |  |  |  |
| Median (IQR) | 64 (58–69) | 64 (53–65) | 64 (57–66) |
| Height, cm |  |  |  |
| Median (IQR) | 175 (168–180) | 175 (170–180) | 175 (168–180) |
| Weight, kg |  |  |  |
| Median (IQR) | 83 (77–91) | 87 (70–110) | 84 (77–95) |
| Comorbidities |  |  |  |
| Hypertension | 11 (44.0%) | 1 (20.0%) | 12 (40.0%) |
| Diabetes | 6 (24.0%) | 0 | 6 (20.0%) |
| Cardiovascular disease | 4 (16.0%) | 0 | 4 (13.3%) |
| Malignancies | 2 (8.0%) | 1 (20.0%) | 3 (10.0%) |
| Cerebrovascular disease | 1 (4.0%) | 0 | 1 (3.3%) |
| Chronic kidney disease | 1 (4.0%) | 0 | 1 (3.3%) |
| Medications at baseline |  |  |  |
| Antiplatelet therapy | 4 (16.0%) | 0 | 4 (13.3%) |
| ACE inhibitors | 2 (8.0%) | 0 | 2 (6.7%) |
| Angiotensin receptor  blocker | 8 (32.0%) | 1 (20.0%) | 9 (30.0%) |
| Antihypertensive therapy | 8 (32.0%) | 1 (20.0%) | 9 (30.0%) |
| Inhaler | 0 | 0 | 0 |
| Insulin | 1 (4.0%) | 0 | 1 (3.3%) |
| Oral anticoagulants | 0 | 0 | 0 |
| Oral antidiabetics | 5 (20.0%) | 0 | 5 (16.7%) |
| Proton pump inhibitors | 0 | 0 | 0 |
| Steroids | 1 | 0 | 1 |
| Signs and symptoms |  |  |  |
| Fever | 22 (88.0%) | 4 (80.0%) | 26 (86.7%) |
| Dry cough | 14 (56.0%) | 2 (40.0%) | 16 (53.3%) |
| Diarrhea | 5 (20.0%) | 0 | 5 (16.7%) |
| Fatigue | 5 (20.0%) | 2 (40.0%) | 7 (23.3%) |
| Myalgia | 4 (16.0%) | 0 | 4 (13.3%) |
| Anorexia | 2 (8.0%) | 0 | 2 (6.7%) |
| Respiratory support upon enrollment |  |  |  |
| Invasive ventilation | 0 | 1 (20.0%) | 1 (3.3%) |
| Continuous positive  airway pressure/non-  invasive ventilation | 25 (100.0%) | 4 (80.0%) | 29 (96.7%) |
| Time to start of ventilation from hospitalization, days |  |  |  |
| Median (IQR) | 2 (1–3) | 3 (3–3) | 2 (1–3) |
| Time from symptom onset to treatment, days |  |  |  |
| Median (IQR) | 8.5 (6.3–12.8) | 13 (12.5–13.5) | 10 (7–13) |
| PP arterial O_2_/fraction inspired O_2_ |  |  |  |
| Median (IQR) | 132.00  (92.86–154.00) | 96.00  (95.00–96.00) | 109.17  (92.86–153.00) |

*ACE* angiotensin-converting enzyme, *IQR* interquartile range, *PP* partial pressure.

**Supplementary Table 2** Baseline laboratory and hematology parameters for siltuximab-treated patients.

| Parameter | Siltuximab-treated patients, IMV (*n* = 5) | Siltuximab-treated patients, CPAP/NIV (*n* = 25) | All siltuximab patients (*N = 30*) |
| --- | --- | --- | --- |
| Hematological parameters,  median (IQR) |  |  |  |
| White blood cells,  median absolute  number/µL (IQR) | 7450 (5500–16 870) | 8550  (6700–10 430) | 8515  (6700–10 470) |
| Hemoglobin, median  g/dL (IQR) | 14.70  (13.40–16.00) | 13.30  (12.60–14.30) | 13.40  (12.60–14.40) |
| Platelets, median  absolute number/μL  (IQR) | 145 000  (140 000–153 000) | 223 000  (167 000–297 000) | 215 000  (147 000–279 000) |
|  | Siltuximab-treated patients, IMV (*n* = 3) | Siltuximab-treated patients, CPAP/NIV (*n* = 22) | All siltuximab patients (*n* = 25) |
| Lymphocytes, median absolute number/μL (IQR) | 860  (720–13,830) | 750  (500–880) | 760  (540–880) |
| Monocytes, median absolute number/μL (IQR) | 430  (360–610) | 285  (190–390) | 310  (200–390) |
|  | Siltuximab-treated patients, IMV (*n* = 5) | Siltuximab-treated patients, CPAP/NIV (*n* = 23) | All siltuximab patients (*n* = 28) |
| Neutrophils, median absolute number/μL (IQR) | 6140 (4180–12 500) | 7240  (5520–9140) | 7020  (5505–9320) |
|  | Siltuximab-treated patients, IMV (*n* = 5) | Siltuximab-treated patients, CPAP/NIV (*n* = 25) | All siltuximab patients (*n* = 30) |
| Laboratory parameters  median (IQR) |  |  |  |
| AST, median U/L (IQR) | 114 (47–114) | 56 (43–77) | 56 (43–89) |
| ALT, median U/L (IQR) | 62.0 (51.0–107.0) | 56.0 (33.0–77.0) | 57.5 (33.0–92.0) |
| Creatinine, median mg/dL (IQR) | 0.960 (0.740–1.010) | 0.850 (0.710–1.120) | 0.875 (0.710–1.090) |
|  | Siltuximab-treated patients, IMV (*n* = 2) | Siltuximab-treated patients, CPAP/NIV (*n* = 6) | All siltuximab patients (*n* = 8) |
| Procalcitonin, median ng/mL (IQR) | 0.445 (0.390–0.500) | 0.840 (0.200–3.460) | 0.445 (0.285–2.385) |
|  | Siltuximab-treated patients,  IMV (*n* = 5) | Siltuximab-treated patients,  CPAP/NIV (*n* = 23) | All siltuximab patients (*n* = 28) |
| Lactate dehydrogenase, median U/L (IQR) | 520.0 (390.0–579.0) | 500.0 (382.0–565.0) | 505.5 (384.0–567.0) |

*ALT* alanine aminotransferase, *AST* aspartate aminotransferase, *CPAP* continuous positive airway pressure, *IMV* invasive mechanical ventilation, *IQR* interquartile range, *NIV* non-invasive mechanical ventilation.

**Supplementary Table 3** Proportion successful prediction of outcome.

|  | | | Predicted status | |  |
| --- | --- | --- | --- | --- | --- |
| Outcome parameter | Day of covariate measurement | Actual status | Dead or same/deterioration | Alive or improvement | Probability correct |
| Day 30 mortality | 1 | Dead | . | 4 | 0.82 |
|  |  | Alive | . | 18 | . |
|  | 4 | Dead | 2 | 2 | 0.86 |
|  |  | Alive | 1 | 17 | . |
| Day 30 ventilatory status | 1 | Same/deterioration | . | 9 | 0.59 |
|  |  | Improvement | . | 13 | . |
|  | 4 | Same/deterioration | 8 | 1 | 0.91 |
|  |  | Improvement | 1 | 12 | . |

**Supplementary Table 4** Summary of adverse events in siltuximab-treated patients.

|  | Siltuximab-treated patients, IMV (*n* = 5) | Siltuximab-treated patients, CPAP/NIV (*n* = 25) | All siltuximab patients (*N = 30*) |
| --- | --- | --- | --- |
| At least one TEAE | 5 (100.0%) | 21 (84.0%) | 26 (86.7%) |
| At least one SAE | 2 (40.0%) | 9 (36.0%) | 11 (36.7%) |
| At least one severe TEAE | 5 (100.0%) | 21 (84.0%) | 26 (86.7%) |
| Deaths | 2 (40.0%) | 8 (32.0%) | 10 (33.3%) |
| At least one AE of CTCAE grading Mild | 0 (0.0%) | 3 (12.0%) | 3 (10.0%) |
| At least one AE of CTCAE grading Moderate | 2 (40.0%) | 11 (44.0%) | 13 (43.3%) |
| At least one AE of CTCAE grading Severe | 4 (80.0%) | 20 (80.0%) | 24 (80.0%) |
| At least one AE of CTCAE grading Life Threatening | 2 (40.0%) | 4 (16.0%) | 6 (20.0%) |
| At least one AE of CTCAE grading Death | 2 (40.0%) | 8 (32.0%) | 10 (33.3%) |

*AE* adverse event, *CTCAE* Common Terminology Criteria for Adverse Events, *SAE* serious adverse event, *TEAE* treatment-emergent adverse event.

**Supplementary Table 5** Summary of adverse events of special interest stratified by grade (siltuximab-treated patients).

|  |  | CTCAE Grade | | | | |
| --- | --- | --- | --- | --- | --- | --- |
| System organ class | Preferred term | 1 | 2 | 3 | 4 | 5 |
| Patients with at least one event |  | 2 (6.7%) | 13 (43.3%) | 24 (80.0%) | 6 (20.0%) | 10 (33.3%) |
| Investigations |  | 0 | 0 | 13 (43.3%) | 0 | 0 |
|  | Alanine aminotransferase increased | 0 | 0 | 12 (40%) | 0 | 0 |
|  | Aspartate aminotransferase increased | 0 | 0 | 3 (10%) | 0 | 0 |
|  | Lipase increased | 0 | 0 | 1 (3.3%) | 0 | 0 |
|  | Platelet count decreased | 0 | 0 | 1 (3.3%) | 0 | 0 |
| Infections and infestations |  | 0 | 2 (6.7%) | 8 (26.7%) | 2 (6.7%) | 1 (3.3%) |
|  | Bacterial sepsis | 0 | 0 | 4 (13.3%) | 0 | 0 |
|  | Pneumonia, bacterial | 0 | 0 | 2 (6.7%) | 0 | 0 |
|  | Urinary tract infection | 0 | 2 (6.7%) | 0 | 0 | 0 |
|  | Bronchopulmonary aspergillosis | 0 | 0 | 1 (3.3%) | 0 | 0 |
|  | Encephalitis, viral | 0 | 0 | 1 (3.3%) | 0 | 0 |
|  | Pneumonia | 0 | 0 | 1 (3.3%) | 1 (3.3%) | 0 |
|  | Pneumonia, *Pseudomonas* | 0 | 0 | 2 (6.7%) | 0 | 0 |
|  | Septic shock | 0 | 0 | 0 | 1 (3.3%) | 1 (3.3%) |
| Vascular disorders |  | 0 | 2 (6.7%) | 7 (23.3%) | 1 (3.3%) | 0 |
|  | Hypertension | 0 | 1 (3.3%) | 5 (16.7%) | 0 | 0 |
|  | Hypotension | 0 | 0 | 2 (6.7%) | 1 (3.3%) | 0 |
|  | Peripheral artery hematoma | 0 | 1 (3.3%) | 0 | 0 | 0 |
| Respiratory, thoracic, and mediastinal disorders |  | 2 (6.7%) | 2 (6.7%) | 6 (20.0%) | 1 (3.3%) | 8 (26.7%) |
|  | Respiratory failure | 0 | 0 | 0 | 0 | 8 (26.7%) |
|  | Pulmonary embolism | 0 | 1 (3.3%) | 3 (10.0%) | 1 (3.3%) | 0 |
|  | Pneumomediastinum | 1 (3.3%) | 0 | 0 | 0 | 0 |
|  | Pneumothorax | 1 (3.3%) | 1 (3.3%) | 2 (6.7%) | 0 | 0 |
|  | Pulmonary hemorrhage | 0 | 0 | 1 | 0 | 0 |
| Psychiatric disorders |  | 0 | 5 (16.7%) | 3 (10.0%) | 0 | 0 |
|  | Delirium | 0 | 4 (13.3%) | 3 (10.0%) | 0 | 0 |
|  | Confusional state | 0 | 1 (3.3%) | 0 | 0 | 0 |
| Renal and urinary disorders |  | 0 | 0 | 5 (16.7%) | 1 (3.3%) | 0 |
|  | Acute kidney injury | 0 | 0 | 4 (13.3%) | 1 (3.3%) | 0 |
|  | Hematuria | 0 | 0 | 1 (3.3%) | 0 | 0 |
| Gastrointestinal disorders |  | 0 | 0 | 3 | 0 | 0 |
|  | Small intestinal hemorrhage | 0 | 0 | 2 (6.7%) | 0 | 0 |
|  | Gastric hemorrhage | 0 | 0 | 1 (3.3%) | 0 | 0 |
| Skin and subcutaneous tissue disorders |  | 0 | 2 (6.7%) | 0 | 0 | 0 |
|  | Rash, maculo-papular | 0 | 2 (6.7%) | 0 | 0 | 0 |
| Blood and lymphatic system disorders |  | 0 | 0 | 0 | 1 (3.3%) | 0 |
|  | Disseminated intravascular coagulation | 0 | 0 | 0 | 1 (3.3%) | 0 |
| Cardiac disorders |  | 0 | 1 (3.3%) | 0 | 1 (3.3%) | 0 |
|  | Atrial fibrillation | 0 | 1 (3.3%) | 0 | 0 | 0 |
|  | Bradycardia | 0 | 0 | 0 | 1 (3.3%) | 0 |
| General disorders and administration site conditions |  | 0 | 1 (3.3%) | 0 | 0 | 1 (3.3%) |
|  | Hypothermia | 0 | 1 (3.3%) | 0 | 0 | 0 |
|  | Multiple organ dysfunction syndrome | 0 | 0 | 0 | 0 | 1 (3.3%) |
| Nervous system disorders |  | 0 | 1 (3.3%) | 2 (6.7%) | 0 | 0 |
|  | Peripheral motor neuropathy | 0 | 0 | 1 (3.3%) | 0 | 0 |
|  | Transient ischemic attack | 0 | 1 (3.3%) | 0 | 0 | 0 |
|  | Cerebrovascular accident | 0 | 0 | 1 (3.3%) | 0 | 0 |

*CTCAE* Common Terminology Criteria for Adverse Events.
